# Deep reinforcement learning for microstructural optimisation of silica aerogels

**DOI:** 10.1038/s41598-024-51341-y

**Published:** 2024-01-17

**Authors:** Prakul Pandit, Rasul Abdusalamov, Mikhail Itskov, Ameya Rege

**Affiliations:** 1 Department of Aerogels and Aerogel Composites, Institute of Materials Research, German Aerospace Center, Linder Höhe, 51147 Cologne, NRW Germany; 2 Department of Continuum Mechanics, RWTH Aachen University, Eilfschornsteinstr. 18, 52062 Aachen, NRW Germany; 3 School of Computer Science and Mathematics, Keele University, Keele, Staffordshire ST5 5BG UK

**Keywords:** Mechanical engineering, Materials science, Computational science

## Abstract

Silica aerogels are being extensively studied for aerospace and transportation applications due to their diverse multifunctional properties. While their microstructural features dictate their thermal, mechanical, and acoustic properties, their accurate characterisation remains challenging due to their nanoporous morphology and the stochastic nature of gelation. In this work, a deep reinforcement learning (DRL) framework is presented to optimise silica aerogel microstructures modelled with the diffusion-limited cluster–cluster aggregation (DLCA) algorithm. For faster computations, two environments consisting of DLCA surrogate models are tested with the DRL framework for inverse microstructure design. The DRL framework is shown to effectively optimise the microstructure morphology, wherein the error of the material properties achieved is dependent upon the complexity of the environment. However, in all cases, with adequate training of the DRL agent, material microstructures with desired properties can be achieved by the framework. Thus, the methodology provides a resource-efficient means to design aerogels, offering computational advantages over experimental iterations or direct numerical solutions.

## Introduction

Silica aerogels have a fascinating history dating back to 1930’s, when the first ‘aerogel’ was synthesised by Kistler^1^. This pioneering work in the synthesis of these porous materials, in which the liquid phase is extracted from the pores of a gel, laid the foundation for the development of various types of aerogels, which are now considered to be among the lightest solid materials in use. For example, silica aerogel is synthesised by drying a silica gel and is characterised by very low density ($<0.20 \,\hbox {g/cm}^{3}$),high porosity (up to ${99.98}\,{\hbox {v}/\hbox {v}}$), low thermal conductivity (up to ${0.01 }\,\hbox {W}\,\hbox {m}^{-1}\,\hbox {K}^{-1}$), and sound velocity as low as ${20.00}\,{\hbox {m\,s}^{-1}}$^2^. Due to such exceptional properties, the properties and potential applications of silica aerogels, including but not limited to thermal insulation, stardust collection, packaging, and biomedicine have intensively been explored^3,4^. These features, ranging from thermal conductivity to diverse mechanical and optical properties, are strongly influenced by the microstructure of the material, such as pore size distribution, particle connectivity, and specific surface area^5–7^. Traditional aerogel synthesis relies on recipes or trial-and-error approaches based on chemical principles, which sometimes limits the exploration of new and sustainable synthesis routes. This is also compounded by the additional lack of a comprehensive understanding of the complex structure–property relations in aerogels, making it difficult to predict the behaviour of these materials under different environmental and loading conditions. Therefore, the ability to optimise the microstructure of silica aerogels through targeted synthesis and, in turn, to tailor their properties for a given application remains a significant challenge.

In the last decade, deep learning and artificial intelligence (AI) approaches have gained significant attention in several domains such as image processing^8^, optimisation problems^9^, or even general game playing^10^. Recently, the influence of AI has also spread to the natural sciences, especially engineering, physics, materials science, or even chemistry^11^. In particular, large amount of data makes it possible to pursue new approaches, e.g., the prediction of protein folding^12^, the description of material behaviour^13–15^, microstructure optimisation^16,17^, or even the discovery of materials with novel properties^18–20^. Moreover, in the field of porous materials, data-driven approaches have been utilised also for rapid material characterisation, to enable accelerated materials synthesis^21,22^. In addition to such data-driven applications, reinforcement learning is being utilised to develop self-learning laboratories for autonomous exploration of advanced materials^23^. This aspect of designing and developing novel materials leads to huge opportunities for the future. Moreover, the application of deep learning is limited not only to materials but has also been applied in the development of meta-materials. Recently, deep learning methods for the structure–property map inversion of truss meta-materials have been reported^24^. In the context of meta-materials with nature-inspired architectures, an unsupervised generative adversarial model was developed to design, model, and print 3D structures for diverse applications^25^.

**Figure 1:**
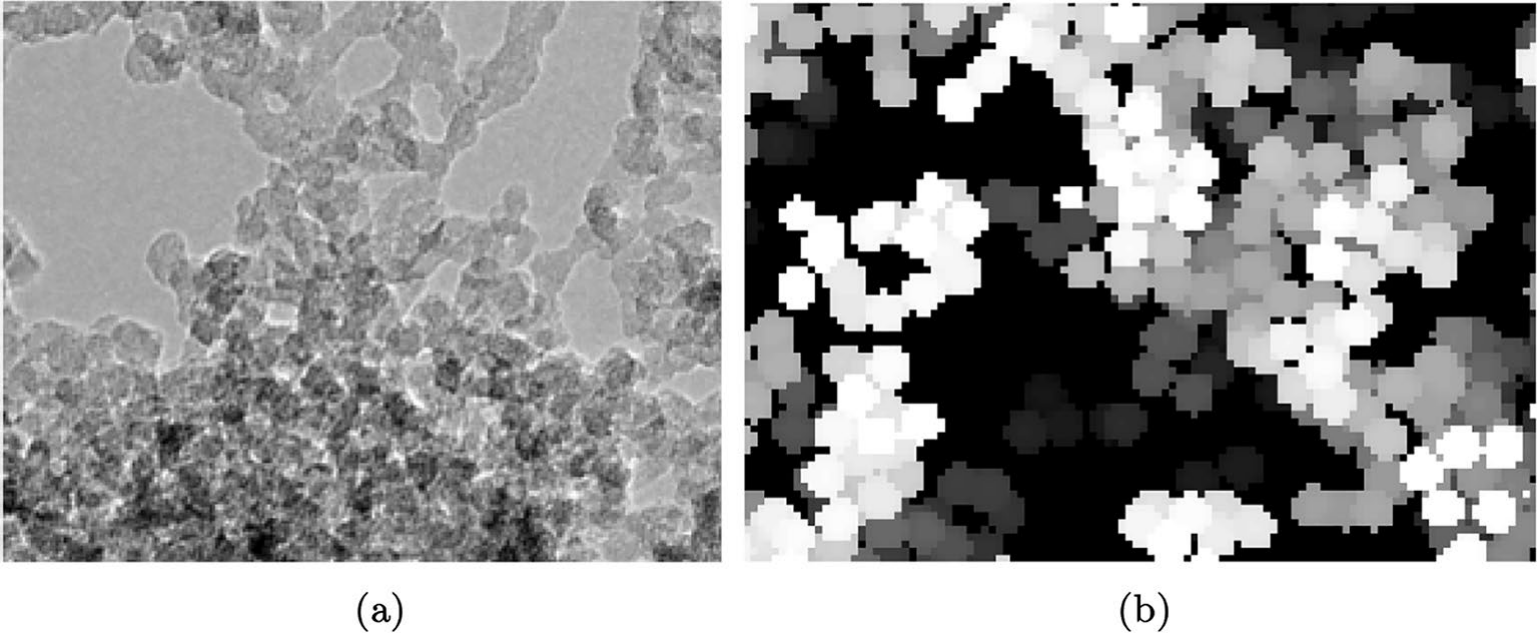
(**a**) A transmission electron microscopy (TEM) image of silica aerogel from^26^. (**b**) Voxelised computational reconstruction from DLCA models to show the DLCA microstructure morphology in 2D.

With the aim of describing the complex structure-property relations in silica aerogels, several reports on their computational modelling have been communicated in the literature, particularly with, molecular dynamics^27–30^, coarse-grained simulations^31–33^ as well based on tomographic imaging through CT-scans^34,35^. A computational model can be defined to be a digital twin of any material, only when the model accurately describes the physical and chemical characteristics on a multi-dimensional scale. Thus, it is essential to model not only the morphology but also the chemical kinetics of the formation of the gelled structure. On a coarse-grained scale of silica aerogels, the diffusion limited cluster–cluster aggregation (DLCA) algorithm has been shown to effectively model the structural morphology^32^ and also their mechanical behaviour^31^. For example, in Fig. 1a a hierarchical clustered network morphology of a silica aerogel sample observed under a scanning electron microscope (SEM) is represented. Figure 1b visualises its corresponding computational parallel, generated through stacking of 2D slices of DLCA structures. Moreover, it has also been shown to qualitatively describe the gelation kinetics post the nucleation of the secondary particles during the sol–gel process^36,37^. Thus, the DLCA models, if optimised, present the opportunity to be extrapolated for the controlled synthesis of silica aerogel in an automated deep-learning laboratory, while expediting the process and facilitating the discovery of more efficient and sustainable aerogel synthesis pathways.

All modelling approaches have a common process characteristic: they require considerable computation times. Therefore, it is a major computational challenge to investigate how certain input parameters influence the mechanical properties of the generated aggregated structures. Usually, to this end, an exhaustive search is performed, to generate a tailored structure to fit the desired properties. Machine learning (ML) approaches can significantly contribute to improving this process by acting as surrogate models for property predictions. Different ML modes such as support vector machine (SVM)^38,39^, random forest^40,41^, convolutional neural networks (CNNs)^42–44^, multi-layer perceptrons (MLP)^45^, and graph-based neural networks^46–48^ have been adopted as surrogate models to relate the microstructures or microstructural features into the material properties in many applications. In the context of silica aerogels, as an extension of the inversed design method presented in^49^, this work explores the combination of random forests with reinforcement learning for performing microstructural optimisation of silica aerogels. Thereby, a deterministic type agent performs actions in the design environments to achieve a maximum reward. By specifying the inputs of the DLCA method as actions and the fractality as well as the mechanical properties as states, a tailored aggregated structure can be generated. However, here the key question arises as to which of the several properties of silica aerogels should be optimised. While silica aerogels are primarily investigated as thermal insulators, choosing thermal conductivity would be the quintessential choice. However, it has been thoroughly investigated in the past decades. What remains fascinating is to simultaneously improve their mechanical characteristics. In this work, the main objective is to investigate their elastic modulus (E) as well as their fractal characteristics, based on fractal dimension $(f_d)$. A key objective is to improve the mechanical strength of aerogels by understanding their microstructure, enabling aerogels to withstand loads without collapsing or breaking. The inter particle connectivity of the microstructure has a crucial influence on the overall macroscale behaviour^50^.

The paper is organized as follows. The results of the data generation, the surrogate model prediction of material properties, and also the inverse design with the DRL framework are presented in “Results”. A general discussion and outlook of the research is presented in “Discussion”, followed by the methodology of generating the data and a detailed description of the development and application of the DRL framework in “Methods”.

**Figure 2:**
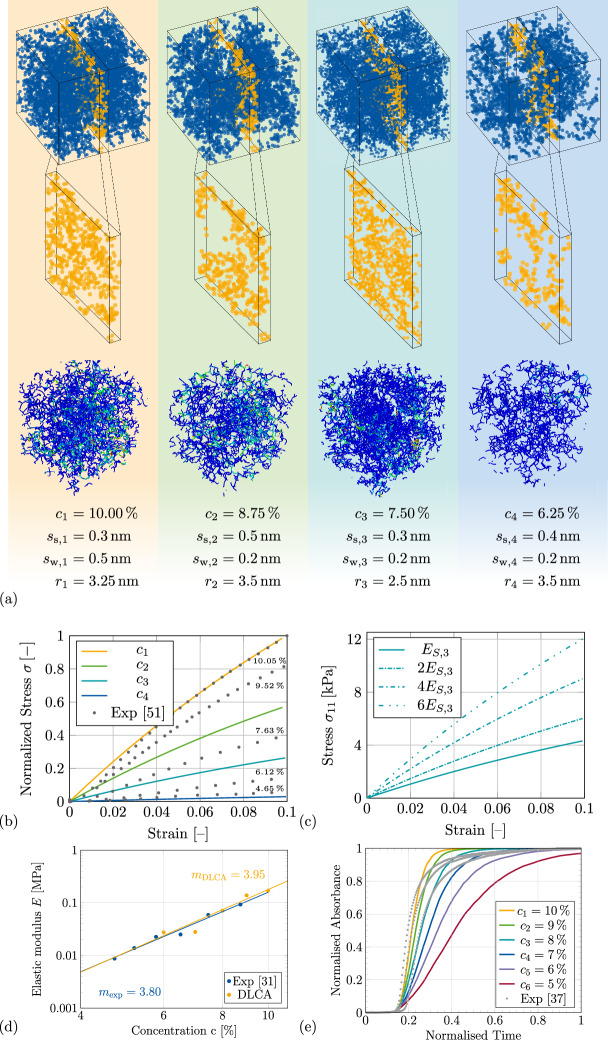
(**a**) Visualisation of four aggregated structures for different concentrations, showing the 3D particles, a visualisation of the cross-section in the center, and the compression tests from FE simulations. In addition, the (**b**) shows the stress–strain response for the four concentrations including a comparison to literature data^51^. The influence of the varying modulus of elasticity of each beam element is also shown for the aggregated structure of concentration $c_3$ in (**c**). The comparison of the scaling exponent of the elastic modulus from the DLCA computational results to the experimental data^31^ in (**d**) and the gelation kinetics of DLCA to experimental data^37^ in (**e**) are visualised.

## Results

This section presents the data generation through DLCA algorithm and its pre-processing results as well as results on the microstructure optimisation with DRL.

**Figure 3:**
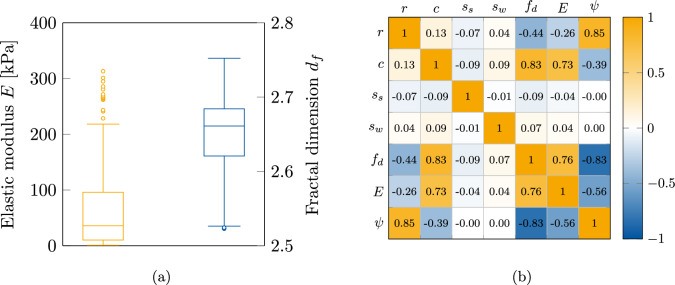
(**a**) Boxplot visualisation of the training dataset’s target properties E and $f_d$ after data pre-processing. (**b**) Correlation matrix between the particle radius *r*, the concentration *c*, the seed step size $s_{\text {s}}$, the walker step size $s_{\text {w}}$, the fractal dimension $f_{d}$, the elastic modulus *E* and the graph network density $\psi$.

### Data generation and analysis

In the present study, for the development of the DRL framework, a total of 1708 aggregates were generated using the DLCA algorithm developed in Matlab. To analyze their mechanical response, these aggregated microstructures were simulated under ${10.00}{\%}$ compressive strain in Abaqus. More details about the finite element simulations can be found in “Finite elements simulations of representative volume elements”. While the initial pool of aggregates was larger, convergence errors in conjunction with shorter elements along with the lack of formation of a backbone structure resulted in only realising 1708 meaningful simulations. This was an expected outcome, due to the randomness involved in the DLCA algorithm and the wide range of inputs provided for the generation of the data set. This resulted in structures with fewer cluster–cluster interactions, leading to localised cluster formation, which in turn led to the absence of backbone formation in certain cases. The representative volume elements (RVE)s lacking backbones are non-physical and were therefore not considered for the training data, as silica aerogels always exhibit a load-bearing backbone when exposed to compressive forces. Exemplary aggregated structures for the concentrations of $c_1 = {10.00}{\%}$, $c_2 = {8.75}{\%}$, $c_3 = {7.50}{\%}$ and $c_4 = {6,25}{\%}$ are depicted in Fig. 2a. Here, *r* denotes the particle radius, $s_{\text {s}}$ and $s_{\text {w}}$ are the step sizes of seeds and walkers. The general 3D network of the aggregated structure together with a cross-section visualisation is shown in the center. Additionally, the RVE compression simulations up to ${10.00}{\%}$ are visualised along with their mechanical response for the different concentrations. Figure 2b demonstrates the influence of the concentration on the mechanical behaviour. It can be observed that increasing the concentration enhances the stiffness of the aerogel model. This seems reasonable enough following the scaling behaviour observed typically in porous materials. Furthermore, the influence of skeletal Young’s modulus is visualised for the aggregated structure of concentration $c_3$ (see Fig. 2c). It can be seen that the stiffness of the beam elements increases directly in a linear fashion with increasing $E_{S,3}$. The validation of the beam element Young modulus can be performed experimentally by calculating skeletal Young’s modulus of the silica aerogel. This increase is justified since amongst the three modes of deformation, bending contributes at most to stresses in silica aerogels and within the elastic regime, the bending stress is proportional to Youngs’s modulus as shown by Ma et al.^52^.

As discussed in “Introduction”, the DLCA model can be defined as a digital twin only after multi-dimensional validation of the material morphology and chemical kinetics. To this extent, the Fig. 2d describes the validation of the scaling exponent of the elastic modulus of the silica aerogel microstructure modelled with DLCA algorithm. The exponent of 3.95 (calculated for different concentrations of aggregates with $r={3.00}\,\hbox {nm}$, $s_{\text {s}} = {0.30}\,\hbox {nm}$ and $s_{\text {w}} = {0.30}\,\hbox {nm}$) in this study closely aligns with the exponent of 3.80 obtained from experimental measurements. Moreover, it can be seen in Fig. 2e, that the DLCA gelation kinetics qualitatively describes the post-nucleation sol-gel growth of the structure. For the computational model, these kinetics is influenced by the step size of the walkers (explained in “Methods”): the higher the step size, the faster the growth. Thus the step size is a numerical construct of the influence of temperature on the secondary particles.

The data set generated from DLCA and its mechanical simulations was cleaned to remove variance for training of the property predictor. The variance is introduced into the system due to the randomness involved in the DLCA process. As such, the converged 1708 samples were averaged over each sample, thus leading to a final data set of 200 structures. The training data set is described with a box plot in Fig. 3a. It was observed that the averaging helps to remove several outliers from the training data set, thus enabling accurate regression analysis by the surrogate model.

To understand the correlation between the DLCA parameters and material properties of the generated aggregates as well as the mechanical response, a correlation matrix was calculated, and is illustrated in Fig. 3b. It is immediately apparent that both particle step sizes have nearly no effect on any other parameter. This can be explained by the fact that the step size is a mathematical construct to model the influence of temperature in the gelation process, and as such it only affects the time of gelation in sol-gel processes^53^. Note that the particle position is additionally adjusted during aggregation to prevent particle overlap. This is a helpful step to avoid very small elements, such that the distance of the aggregated particles is exactly equal to the sum of the radii. As already known from literature there exists a positive correlation between the elastic modulus *E* and the concentration *c*^54^, which can be itself validated from the correlation matrix. The concentration has also a positive correlation with the fractality. Furthermore, the analysis of the matrix reveals an inverse relationship between the radius and the elastic modulus. One other interesting observation is that the correlation between the elastic modulus *E* and the graph network density $\psi$ is negative. This leads to the assumption that as the number of network connections increases, the number of cluster localisations grows, implying that fewer particles can actually contribute to the backbone structure.

**Figure 4:**
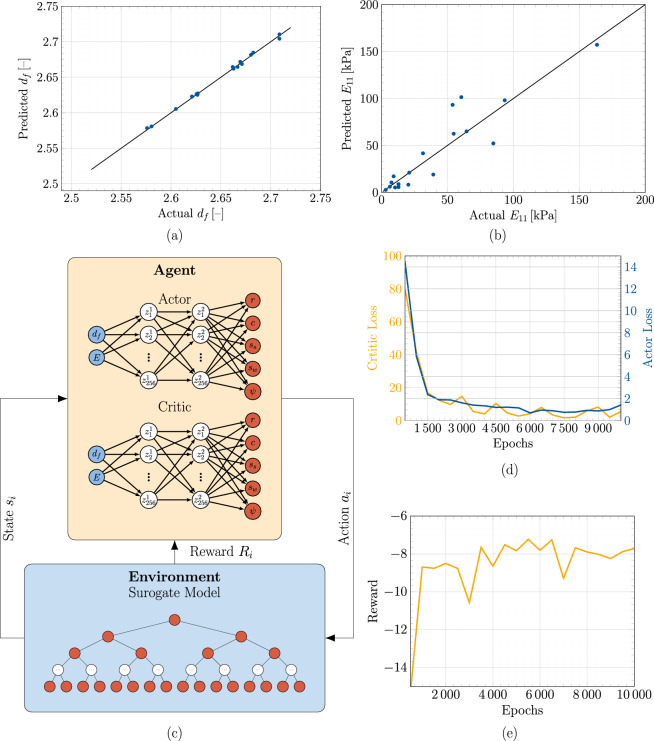
(**a,b**) Comparison of predicted vs actual material properties for the random forest model over the test set. (**c**) Schematic visualisation of the DRL framework with an agent trained over the environment. (**d**) The actor and the critic loss during the training process of the DRL agent in the ‘low-complexity’ environment. (**e**) Mean episodic award over the training.

### Microstructure optimisation with DRL framework

To reduce computational resources utilised, a surrogate model/property predictor was developed to replace the finite element method (FEM) simulations while performing the inverse design in the DRL framework. The pre-processed data as discussed in “Data generation and analysis” was utilized to train a random forest algorithm as the surrogate model for predicting the material properties directly from the DLCA parameters and the graph density. The pre-processed data was split into training (${90.00}{\%}$) and testing sets (${10.00}{\%}$), so as to avoid information leak during normalisation from the training set to the data set utilised for validation. These sets were then individually normalised between (− 1,1). The property predictor was evaluated on the basis of the mean absolute error (MAE) metrics. To achieve the best metric, the hyper-parameters were tuned with a ‘brute-force’ approach. The trained surrogate model was validated on the test data set and had a $R^2$ score of 0.94. These validation results over the test set can be seen in Fig. 4a,b. Further details of the random forest architecture are discussed in “Numerical surrogate environment”.

**Figure 5:**
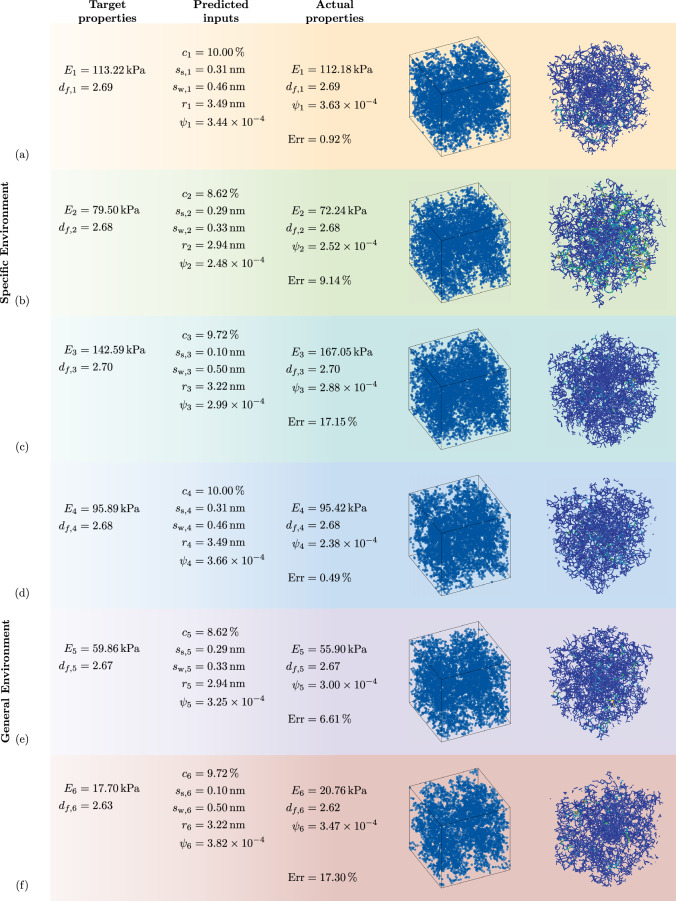
Optimisation of the microstructures with DRL and their validation: visualisation of some selective optimised structures based on desired target properties in the (**a–c**) specific-defined ‘low-complexity’ environment and (**d–f**) in the generalised ‘high-complexity’ environment. To validate the results through the RL approach the specified DLCA algorithm inputs were determined and the resulting structures for both environments were validated by microstructure generation with DLCA and their subsequent FEM analysis.

The deep deterministic policy gradient (DDPG) algorithm^55^ coded in the Stablebaselines3 framework^56^ was utilised to perform the inverse microstructure optimisation over a custom DLCA environment (the details of the environment along with the reward function shaping is explained in “Reinforcement learning framework”). The schematic representation of the DDPG agent working in combination with the environment for the DRL framework can be seen in Fig. 4c. The DRL inverse optimisation problem was tested with respect to two difficulty levels: the ‘low-complexity scenario’, wherein the DDPG model was trained over a single combination of target material features and a ‘high-complexity scenario’ wherein the trained DDPG model was trained over different target material features possible in the production scenario in each episode to achieve generalisability during the production cycle. As compared to a single target learning problem, this increases the complexity involved in learning the Q-function, however, enables the deployment of a single agent for synthesising silica aerogels with diverse properties. In both environments, the DDPG agent learns a policy to maximise the average episodic reward to solve the environment (see “Reinforcement learning framework” for more details). For the ‘low-complexity’ training case, the environment was defined to have material property constant over all episodes. This enabled faster training of the agent to achieve optimised structures at the expense of generalisability. To achieve the optimal inverse design with the DDPG agent in this environment, the DDPG hyperparameters were tuned with Optuna over 100 trials for different combinations. The agent hyperparameters were selected based on the highest episodic mean reward received. The critic and actor loss of the DDPG agent based on the best-selected hyperparameters in the low-complexity environment are shown in Fig. 4d. Figure 4e describes the average episodic reward for the ‘low complexity’ environment. It can be observed that in the initial training epochs, the agent achieves negative rewards based on the reward function defined (explained in “Numerical surrogate environment”). However, as it learns from its experience by interacting with the environment over the training time, the reward increases gradually towards the maximum, indicating that the agent can take actions (DLCA parameters) to solve the provided environment for the desired states ($$E, f_d)$$. Thus these achieved actions from the trained agent at the end of training, provide the user with the optimised microstructure for the desired material properties. To validate the results from the DRL framework, the microstructures with returned actions were regenerated with DLCA and analysed with FEM. In Fig. 5a–c, selective results of the output of the optimised microstructure based on the constant target property environment are presented. It can be observed that the agent effectively optimises the silica aerogel microstructure and predicts the exact DLCA model parameters which would provide the target properties. These structures are accurate to an MAE of about 1.00$${\%}$$ for cases existing in the training data, however on interpolating the material features and as such exploring material properties unseen by the framework during the training phase, results in microstructures with average MAE of approx 10.00$${\%}$$. The training over single material targets allows quicker training times and easy deployment, as accurate optimisations can be achieved after training the agent for 10,000 epochs. Due to the single material property optimised, the episodic length remains constant, which simplifies the learning process. However, to optimise silica aerogels with diverse properties, retraining the agent in a similar environment but with different targets would be necessary.

To solve this problem of retraining the agent each time, a second environment for material optimisation was developed. What differentiates these environments is the definition of the target properties (more details in “Methods”). For each episode, the agent learns how to achieve a different material property, wherein these material properties are sourced from all the possible combinations possible in the material production and can be inputted by the user, thus making it a ‘higher-complexity’ problem. Due to this increase in the complexity of the environment, the agent requires more training epochs to achieve convergence. Accordingly, based on the hyper-parameter optimisation with Optuna, the agent converged with highest mean episodic reward after training for 125,000 epochs. Moreover, the length of each upside is also not constant due to the switch of the material properties over the training period of each episode. It is important to consider that a higher number of training epochs is required to train the ‘high-complexity’ environment, to get a similar accuracy as the ‘low-complexity’ environment. Figure 5d–f visualises some of the selective structures optimised through the general environment with switching material properties over episodes. For most of the required structures, the RL agent achieves the inverse design by optimising the parameters of the DLCA parameters with an MAE of about 6.00${\%}$. However, it was also observed that certain material properties optimised were in a range of about 15.00–25.00% MAE, which is due to the diverse targets experienced by the agent over the number of episodes. This can mostly be related to the higher variance experienced in the FEM simulations, which in turn required an averaged data set to be utilised for training. This also effects the generalisability of the surrogate model over outlying material targets. This lack of training data persists in the ‘high-complexity’ scenario, wherein the DRL framework is not able to build generalised training history in the replay buffer.

## Discussion

Silica aerogels exhibit an unearthed potential for diverse applications, making their reverse synthesis for targeted applications critical. The computational design of silica aerogels with improved strength is a challenging task owing to the complexities of their microstructure. The DRL framework presented above aims to solve this task. The main goal of this research is to design tailored silica aerogel microstructures by utilising the DLCA algorithm for targeted applications. Previous works by our group have focused on ANN based approaches, to perform an inverse structure–property mapping. However, the problem of non-uniqueness appeared to be non-trivial. To overcome this challenge, reinforcement learning techniques are therefore explored. A deterministic algorithm is trained over two environments of varied complexity. What differentiates a deterministic policy algorithm is that it chooses a single action for each state, while a stochastic policy selects from a probability distribution over actions for each state. For the goal of reverse engineering, the DLCA method in combination with FE simulations is used for the data generation. The generated data provides insight into the correlation between the structural and mechanical parameters. The importance of the correlation between concentration and Young’s modulus known from the literature was affirmed. In addition, an interesting observation regarding the effect of network connectivity on the elastic modulus can be found. Accordingly, localised connections weaken the existing backbone structure. Although trained over a limited data set, the proposed RL framework can identify targeted mechanical and structural parameters. The approach has been tested for two levels of complexity, resulting in an average MAE of 10.00${\%}$ depending upon the complexity of the environment. A key observation is the ability to generalise over a continuous input space from comparatively few data points to obtain satisfactory results. Nevertheless, the current approach of reverse engineering is limited only to the elastic modulus and the fractality of the silica aerogels. Certainly, the extension to thermal and acoustic simulations for tailoring further properties directly be achieved in the basis of existing micromechanical models^57^. To account for potential biases or uncertainties, it remains important to improve the training data set. Potential improvements can also be made by the FE technique. This work illustrates a potential solution for a significant improvement of the mechanical properties of silica aerogels by providing a deeper understanding of the micro structural relations and their influence on macroscale properties. The presented research is a first step towards tailor-developing silica aerogels, with the potential for expansion through the incorporation of digital twins emulating the multi-scale behavior of aerogels. This work in itself must not be seen as a direct tool for reverse engineering silica aerogels. Primarily, this approach opens applications for inverse designing aerogels, using DRL to control synthesis process conditions as actions (such as chemical precursors, acid concentration, and reaction temperature). Beyond microstructure optimisation, the application of DRL extends to metamaterials. In this context, the DRL framework can be trained to predict topological features as actions to attain specific material properties as states. This DRL framework is directly applicable in realising (meta)-material microstructures through manufacturing processes like additive manufacturing and 3D printing. Consequently, it provides a resource-efficient means to design aerogels, offering computational advantages over experimental iterations or direct numerical solutions. This approach has the potential to significantly reduce the time and cost associated with developing novel aerogel material structures.

## Methods

There are two main methodological aspects involved in this work for the optimisation of the silica aerogels: the computational modelling and simulation of aggregated structures and the development of a DRL framework to solve an optimisation problem generating a target microstructural morphology. In “Computational modelling and mechanical simulation of the silica aerogel microstructure”, the generation of the silica aerogel microstructure and the mechanical simulations of the RVEs using FEM are discussed. The fundamentals of DRL using a deterministic policy gradient are presented in “Reinforcement learning framework”.

### Computational modelling and mechanical simulation of the silica aerogel microstructure

An initial data set is required to effectively model the morphological and mechanical properties of the microstructure. The inverse prediction is based on the desired elastic modulus and fractal dimension, reflecting the geometric and mechanical characteristics of silica aerogels. To this end, the DLCA algorithm is utilised to create computational silica aerogel microstructures.

#### Diffusion-limited cluster–cluster aggregation method

**Figure 6:**
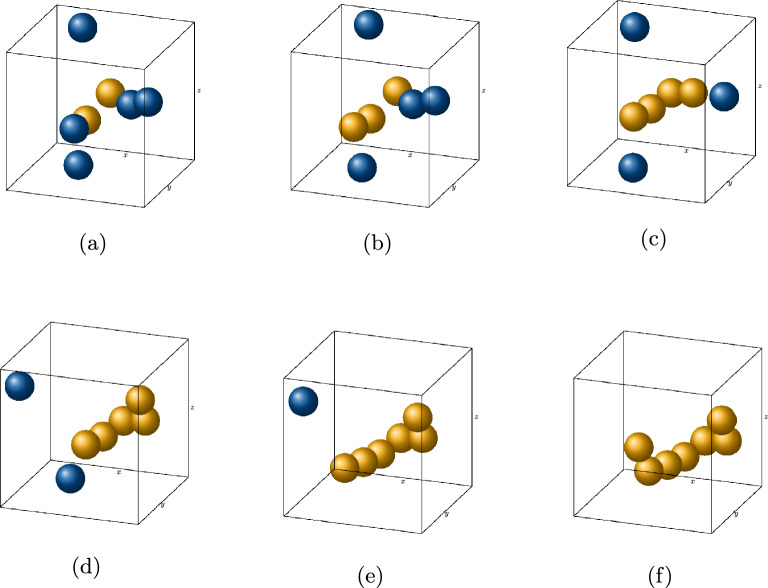
Visualisation of the DLCA algorithm. (**a**) Walkers (blue) and seeds (yellow) initialized in the domain. (**b–e**) Walkers diffuse towards the seeds and aggregate together to form a cluster on reaching a critical distance $\epsilon$. (**f**) Final aggregated structure after all the walkers and seeds connect to form a domain-spanning aggregate.

As discussed in “Introduction”, the DLCA algorithm has been extensively researched for computational modelling of silica aerogel microstructures. Hasmy et al.^32^ showed that structures generated by DLCA matched closely with the small angle neutron scattering (SANS) data for silica aerogels. The starting point of this algorithm is a box with periodic boundary conditions where a total number of $N_\text {all} = N_S + N_W$ of seeds and walker particles are initialized (see Fig. 6 for a 3D visualization). These particles can be placed randomly or in an arranged way within the box. In this example the particles are initialized on random points and they can move freely and periodically according to the random walk theory. For this purpose a seed and walker step size, $s_S$ and $s_W$, are defined. With time, all the walkers diffuse to the seeds and clusters are formed. As soon as two particles of two different clusters exceed a critical distance the two clusters are connected to form one single cluster. The algorithm continues until only one periodically spanning network remains in the system, representing the microstructure of the aerogel. This algorithm was adapted by Abdusalamov et al.^31^ and comparable results in terms of scaling relationships between simulations and experimental results were obtained. A slightly modified DLCA model was developed for this paper as a continuation of the latter work: the random walk process was redefined allowing all particles to move based on a spherical walk. In addition, overlaps of particles are prohibited and corrected at the first aggregation. While this overlap can be considered in the models to describe effects of Oswald ripening^58^ in silica aerogels, their elimination helps to simplify the FEM process and avoid convergence issues. The projection of the particle is adjusted based on the connection vector between two particle positions, ensuring that the connection length equals the sum of both particle radii. The algorithm inputs are the particle radius *r*, the step sizes of seeds $s_{\text {s}}$ and walkers $s_{\text {w}}$, the relative density *c* for a constant RVE domain size of ${200.00}\,\hbox {nm}$. Refer to Fig. 6 for a visualisation of the general process of the DLCA algorithm.

#### Finite elements simulations of representative volume elements

FEM was utilised to describe the mechanical properties of the generated aggregated structures. Based on the DLCA algorithm the particle positions and connections were extracted. In order to perform an FEM simulation, an RVE was generated for each aggregated structure. Thereby, the dimensions of the RVE corresponded to the dimensions of the DLCA simulation box. In addition, periodic boundary conditions were applied^31^. Accordingly, each bounding surface of the RVE was used to generate additional nodes for connections over the boundary. Note that all particles are modeled as nodes, while the connections are exported as beam elements. The simulations are performed in Abaqus  using the linear B31 element formulation based on the Timoshenko beam theory. This formulation guarantees that all three types of bond deformations are considered: bending, torsion, and stretching. Young’s modulus and Poisson’s ratio of each beam are taken have been provided in literature for silica aerogels^33^ with $E_{S} = {10.00}\,\hbox {MPa}$ and $\nu = {0.29}$. For this work, the Young’s modulus of each beam was determined through a correlation to experimental data as $E_{S} = {6.95}\,\hbox {kPa}$. To ensure that compression is symmetric with respect to the centre, additional points in the centre of the RVE are fixed. However, to eliminate convergence issues in the process of data generation, unlike in previous works, the contact between all beam elements is not taken into account. As the RVE is only compressed up to ${10.00}{\%}$, no significant contact between the beams occurs and therefore no effect on the initial stiffness response is noticeable. The simulations are generated as input decks in Matlab  and executed for uniaxial compression in Abaqus. The deformation as well as the force response are exported to analyse the mechanical properties of the aggregated silica aerogel structures.

#### Graph density for microstructural connectivity

Connectivity plays a pivotal role in understanding elastic behaviour of porous materials, offering critical insights into the relationship between microstructure and mechanical properties. Power law exponents $(E \propto \rho ^m)$ describe the connection between the mechanical behaviour of materials and their density. High connectivity within the material significantly influences the elastic modulus and other mechanical attributes. Increased connectivity in the microstructure leads to a lower power exponent due to enhanced load distribution capacity, whereas decreased connectivity results in a higher power law exponent resulting from the formation of backbone paths, indicating greater sensitivity to changes in porosity and structural disorder. Graph theory can be utilised for quantifying the connectivity of porous materials. One such metric is the density of a graph. For a graph, its density ($\psi$) is defined as:

1 $$\begin{aligned} \psi = \frac{2E}{V(V - 1)} \ , \end{aligned}$$

where |*E*| and |*V*| represent the number of edges and nodes in the graph, respectively. For calculation of the graph density and the microstructural connectivity, the particles of the DLCA model were exported as nodes and their respective bonds as edges using the NetworkX package. Incorporating graph density as a quantitative metric enhances the precision of characterising the connectivity-dependent elastic behaviour of porous materials, facilitating comparative analysis between the optimised to desired microstructures.

### Reinforcement learning framework

Reinforcement learning is a closed-loop ML technique that deals with understanding how an agent interacts in an environment (real or simulated) to achieve desired goals. In this process, an intelligent agent performs actions to achieve maximum feedback in the form of a reward. During this process, the agent interacts with the environment trying to adjust, identify and learn the most suitable actions making a trade-off between exploration and exploitation. An RL framework is commonly described based on the Markov decision process and consists of:

- a set of states *s* for states $\mathcal {S}$,
- a set of actions *a* taken for actions $\mathcal {A}$,
- transition probabilities $P( s' \mid s,a)$ from a state *s* to a state $s'$ for an action *a*,
- as well as the reward $\tilde{r}$ for the transition from state *s* to a state $s'$ for an action *a*.

A reward is a measure of how successful the actions of the agent were with respect to the desired state. In this work, a deterministic policy gradient algorithm was employed on a custom DLCA environment and will shortly be discussed in the following section.

#### Deterministic policy gradient algorithms

In general, both deterministic and stochastic policy gradient-based algorithms can be used for continuous action spaces. However, stochastic policies suffer from a major difficulty, which is the lack of computational efficiency due to the computation of a parametric probability distribution responsible for the stochastic selection of actions^59^. Stochastic policy gradients rely on integration over state and action spaces, resulting in a larger number of training samples. In the case of deterministic policies, the integration is only performed over the state space. Although the action space for the inverse mapping is not high dimensional, DDPG allows sufficient exploration of the action space while still learning the deterministic target policy. As stated in^59,60^ the DDPG algorithm consists of two components: an actor and a critic network. The actor learns a deterministic policy $\mu (s)$ that maps states to actions, while the critic learns an action-value function $Q^*(s',a)$ that estimates the expected return of following the actor’s policy from a given state. The action-value function, also called the Q function is calculated as:

2 $$\begin{aligned} Q^*(s,a) = \underset{s' \sim P}{{\mathrm E}}\left[ \tilde{r} + \gamma \max _{a'} Q^*(s', a')\right] \, , \end{aligned}$$

where $\langle \cdot \rangle '$ represents the transition to the next state or action, where $s'$ is sampled by the environment from a probability distribution $P(\cdot \mid s,a)$ and $\gamma$ is the discount factor. Equation (2) is the starting point for learning an approximator to $Q^*(s,a)$. This approximator is often modelled in the form of a neural network with parameters $\phi$, which have to be optimised through the evaluation of two target networks with respect to the mean-squared Bellman error (MSBE). This leads to the evaluation of the target value $y(\tilde{r},s',d)$ given by:

3 $$\begin{aligned} y(\tilde{r},s',d) = \tilde{r} + \gamma (1 - d) \max _{a'} Q_{\phi }(s',a') \, , \end{aligned}$$

where *d* is the boolean indicating whether $s'$ is terminal or not. Based on this calculated target value, the action-value function can be updated as:

4 $$\begin{aligned} \nabla _\phi \frac{1}{\mid \mathcal {B}\mid } \sum _{(s,a,r^*,s',d)\in \mathcal {B}} \left( Q_\phi (s,a) - y(\tilde{r},s',d)\right) ^2 , \end{aligned}$$

which in turn is used to calculate the step-wise policy gradient given by:

5 $$\begin{aligned} \nabla _\phi \frac{1}{\mid \mathcal {B}\mid } \sum _{(s,a,\tilde{r},s',d)\in \mathcal {B}} Q(s,\mu _\theta (s)) , \end{aligned}$$

where $\mathcal {B}$ is the batch of transitions from a replay buffer $\mathcal {D}$. The target network hyperparameters are updated based on the Polyak averaging

6 $$\begin{aligned} \phi _{\text {targ}} \leftarrow \tilde{\rho } \phi _{\text {targ}} + (1 - \tilde{\rho }) \phi , \end{aligned}$$

where $\tilde{\rho }$ is a hyperparameter ranging between 0 and 1.

#### Numerical surrogate environment

The DRL framework consists of two different complexities. The ‘low-complexity’ environment has the same material property over all training episodes, which are provided by the user as an input. While the ‘high-complexity’ environment switches the target material properties at the end of each episode. These material properties are provided by the user and take into account all possible material features that can exist based on the test data set from the surrogate model. The inputs to the DLCA algorithm act as actions within the framework, while the target material properties are the expected states obtained from these actions. These action ranges were as follows: $r \in \left[ 2.5,3.5\right] , \rho \in \left[ 0.05, 0.1\right] , s_s \in \left[ 0.1,0.5\right] , s_w \in \left[ 0.1,0.5\right]$. Furthermore, from the generated aggregate structures we can calculate the graph network density $\psi \in \left[ {1.67}\,\times \,10^{-4},{7.27}\,\times \,10^{-4}\right]$, which act as an additional action to validate the inversely generated microstructures. The graph density gives a measure of the total connections present in the aggregated structure and is calculated as a ratio of the total number of connections by the total number of possible connections between the nodes, wherein each particle is considered as a node and the inter-particle bond as the connection. Due to the sparse nature of bonds formed in DLCA, we observe such low values of $\psi$. We also limit the observation space for the material outputs, i.e. the fractality $d_f \in [2.45,2.75]$ and elastic modulus $E \in [{1.00}\,\hbox {kPa}, {930}\,\hbox {kPa}]$, so that feasible and experimentally validated solutions can be guaranteed.

A random forest is used as a surrogate model to avoid the high computational and time requirements of iterative training with FEM during microstructure optimisation. This surrogate model is separately trained using supervised learning based on offline generated data from the compression simulation of the RVEs. The random forest consists of five input nodes, i.e. the model parameters of the DLCA, the graph density ($\psi$), and two outputs, i.e. the fractal dimension and the elastic modulus. In the context of DRL, we examine a framework that confronts an inverse optimization problem, differentiating between two distinct scenarios, namely the ‘low-complexity’ and ‘high-complexity’ environments. In the ‘low-complexity’ environment, the DDPG agent is tasked with optimizing a fixed set of target material properties $(E, fd)$, as provided by the user. In this setting, the target material properties remain fixed throughout episodes, and the DDPG agent strives to maximize the cumulative reward by optimising the specified material properties over a constant episode length.

In contrast, the ‘high-complexity’ environment presents a more dynamic challenge. Here, the agent is required to optimise a diverse range of target material properties, with each episode introducing a novel set of properties. Unlike the ‘low-complexity’ setting, the target material properties vary from one episode to the next, leading to a more challenging and dynamic task. Furthermore, the episode length can vary depending on the specific target properties, adding an element of unpredictability. This nuanced differentiation between ‘low-complexity’ and ‘high-complexity’ environments within the DRL framework enables the exploration of adaptive solutions for diverse optimisation problems. For training of DDPG on ‘high-complexity’ environment, each episode was set with terminal condition of 700 training epochs or if the average reward achieved per episode was more than − 0.05 (normalised). If the terminal condition is met, the episode resets to a new target material property and the training continues. During these episodes, in both of the environments, the surrogate model predicts the states achieved for a set of actions sampled from the replay buffer at every epoch. To train the RL agent, and to achieve deterministic optimised actions for the desired states, an episodic reward signal based on relative error was defined as:

7 $$\begin{aligned} \text {Err} = \left( \sum _{i=1}^{n} \Big \Vert s_T(f_d, E)- s(f_d,E) \Big \Vert ^2\right) ^{{1}/{2}} \, , \end{aligned}$$

where $s_T(f_d, E)$ is the target material state and the $s(f_d,E)$ is the states predicted by the surrogate model for the set of actions taken.

## Data Availability

The datasets used and/or analysed during the current study available from the corresponding authors on reasonable request.

## Code availability

The codes used and/or analysed during the current study available from the corresponding authors on reasonable request.
